# Characterising Non-Structural Protein NS4 of African Horse Sickness Virus

**DOI:** 10.1371/journal.pone.0124281

**Published:** 2015-04-27

**Authors:** Lizahn Zwart, Christiaan A. Potgieter, Sarah J. Clift, Vida van Staden

**Affiliations:** 1 Department of Genetics, University of Pretoria, Pretoria, South Africa; 2 Deltamune (Pty) Ltd, Lyttelton, Centurion, South Africa; 3 Department of Biochemistry, Centre for Human Metabonomics, North-West University, Potchefstroom, South Africa; 4 Department of Paraclinical Sciences, Faculty of Veterinary Science, University of Pretoria, Onderstepoort, South Africa; University of Alabama at Birmingham, UNITED STATES

## Abstract

African horse sickness is a serious equid disease caused by the orbivirus African horse sickness virus (AHSV). The virus has ten double-stranded RNA genome segments encoding seven structural and three non-structural proteins. Recently, an additional protein was predicted to be encoded by genome segment 9 (Seg-9), which also encodes VP6, of most orbiviruses. This has since been confirmed in bluetongue virus and Great Island virus, and the non-structural protein was named NS4. In this study, *in silico* analysis of AHSV Seg-9 sequences revealed the existence of two main types of AHSV NS4, designated NS4-I and NS4-II, with different lengths and amino acid sequences. The AHSV NS4 coding sequences were in the +1 reading frame relative to that of VP6. Both types of AHSV NS4 were expressed in cultured mammalian cells, with sizes close to the predicted 17–20 kDa. Fluorescence microscopy of these cells revealed a dual cytoplasmic and nuclear, but not nucleolar, distribution that was very similar for NS4-I and NS4-II. Immunohistochemistry on heart, spleen, and lung tissues from AHSV-infected horses showed that NS4 occurs in microvascular endothelial cells and mononuclear phagocytes in all of these tissues, localising to the both the cytoplasm and the nucleus. Interestingly, NS4 was also detected in stellate-shaped dendritic macrophage-like cells with long cytoplasmic processes in the red pulp of the spleen. Finally, nucleic acid protection assays using bacterially expressed recombinant AHSV NS4 showed that both types of AHSV NS4 bind dsDNA, but not dsRNA. Further studies will be required to determine the exact function of AHSV NS4 during viral replication.

## Introduction

African horse sickness virus (AHSV) is the causative agent of African horse sickness (AHS), a severe non-contagious infectious disease of equids with a mortality rate of over 90% in susceptible horses [1]. AHSV is transmitted between infected and susceptible mammalian hosts primarily by haematophagous *Culicoides imicola* midges [2]. AHS is listed as a notifiable disease by the World Organisation for Animal Health (OIE), which impacts the movement of horses to and from affected areas. Although AHS is enzootic in sub-Saharan Africa with annual outbreaks in southern Africa, the disease has emerged in other countries such as Spain, Portugal and Morocco. The recent effects of climate change further increase the risk of virus introduction into Europe, where the insect vector also occurs [3,4]. The disease is currently managed with vaccination.

AHSV belongs to the genus *Orbivirus* (family *Reoviridae*) and shares many similarities with the orbivirus type species bluetongue virus (BTV), which has been studied more extensively [5]. There are nine known serotypes of AHSV [6,7]. The AHSV genome consists of ten double-stranded RNA segments [8]. As in the case of BTV, it has been widely accepted for decades that each of the ten AHSV genome segments (Seg-1 to Seg-10) contains a single open reading frame (ORF), thereby encoding the seven structural proteins (VP1—VP7) that form the virion, and three non-structural proteins (NS1—NS3/NS3A) with other functions during viral replication [9].

The structure of the AHSV virion is very similar to that of BTV; virions are triple-layered icosahedrally symmetric particles. Major structural proteins VP2 and VP5 comprise the outermost capsid layer of the virion, enclosing the double-layered core particle [10,11]. The core surface layer is composed of VP7 trimers, while the innermost shell is a lattice of VP3 dimers. Inside this shell, the ten genome segments are arranged in three layers of RNA that surround the transcription complexes [12–14] consisting of the three minor proteins VP1 (polymerase), VP4 (capping enzyme) and VP6 (helicase) [15–17]. Viral replication and assembly of new core particles occur in cytoplasmic virus inclusion bodies (VIB) that are formed by non-structural protein NS2 [18,19]. The trafficking and release of mature virus particles from the cell is mediated by NS3, the only membrane-associated viral protein [20–23]. Non-structural protein NS1 self-assembles into tubules of unknown function within the cytoplasm [24,25]. NS1 may also be involved in virus egress and morphogenesis [26], and it was recently shown that NS1 preferentially up-regulates viral protein expression during infection [27].

Seg-9 of AHSV encodes the minor structural protein VP6 [28], as is the case for BTV. VP6 binds dsRNA and other nucleic acids, and functions as the viral helicase [17,29]. Recent bio-informatic analysis suggested that most orbiviruses, including AHSV, contain an additional ORF within the VP6 cistron [30]. This ORF was present in all the analysed Seg-9 sequences of midge-borne and tick-borne orbiviruses with mammalian hosts [30,31], but could not be identified in St Croix river virus (SCRV) [30]. So far, SCRV does not have any known vertebrate host, and could be considered a true ‘tick virus’ [31]. The additional Seg-9 ORF produces a fourth non-structural protein termed NS4 [32]. The predicted NS4 protein was highly conserved among 24 BTV serotypes [33], and in cells infected with BTV or the tick-borne Great Island virus (GIV), NS4 was shown to localise to the nucleus, cytoplasm and cell membrane [32]. The N-terminal basic domain of BTV NS4 may play an important role in its nuclear localisation. In the nucleus, BTV and NS4 strongly co-localised with a nucleolar marker [32,33]. Furthermore, BTV NS4 binds dsDNA, whilst GIV NS4 binds both dsDNA and dsRNA *in vitro* [32]. Interestingly, by using NS4-deletion mutants generated by reverse genetics, BTV NS4 was shown to be dispensable for viral replication, both *in vitro* and *in vivo* [33]. The exact function of NS4 during the replication cycles of these viruses has not yet been defined, but BTV NS4 appears to modulate the host innate immune response to infection and to counteract the cellular antiviral response in interferon-treated cells infected with BTV-8 [33].

Previous analysis of Seg-9 sequences from two AHSV serotypes (AHSV-3 and AHSV-6) showed a high level of conservation, with 97% Seg-9 nucleotide identity and 95% VP6 amino acid identity [28]. Based on three available AHSV Seg-9 sequences, the AHSV NS4 protein was estimated to be about 17–20 kDa in size, with three potential overlapping nuclear localisation signals (NLS) near the N-terminus that predicted dual nuclear and cytoplasmic localisation. Furthermore, AHSV NS4 sequences showed similarity to domains of unknown function (DUFs) containing helical structures associated with putative nucleic acid binding or modifying activity [30,32].

The putative NS4 protein of AHSV has not yet been characterised. Understanding the function of this nuclear protein produced by a cytoplasmic virus could provide valuable new insight into the replication strategy. The aim of this study was to determine whether all nine AHSV serotypes encode NS4, to confirm the expression and investigate the intracellular localisation of AHSV NS4 *in vitro* in mammalian cells and *in vivo* in horses, and to investigate whether it has the nucleic acid binding properties predicted by *in silico* analysis. This is the first study to characterise the fourth non-structural protein of AHSV.

## Materials and Methods

### Cell cultures

BSR cells (derived from BHK21 cells [34]) were a generous gift from Prof Piet van Rijn (Central Veterinary Institute of Wageningen, The Netherlands). Cells were cultured in Eagle’s Minimal Essential Medium (EMEM, Lonza) supplemented with 5% foetal calf serum, non-essential amino acids, antibiotics and antifungals (60 mg/ml penicillin, 60 mg/ml streptomycin, 150 μg/ml fungizone, Highveld Biological) at 37°C with 5% CO_2_ and 90% humidity. Confluent cell monolayers were infected with AHSV at a multiplicity of infection (MOI) of 0.1 to 3 pfu/cell. After incubation at 37°C for an hour, the inoculum was removed and replaced with serum-free EMEM, and the incubation continued under the same conditions for the required time.

### Viruses and sequence analyses

The different AHSV strains used for this study are listed in Table 1. The genome sequences of all viruses were determined essentially as described previously [35]. A separate report on the full genome sequences of these viruses will be submitted for publication. The GenBank accession numbers for the Seg-9 nucleotide sequences are provided.

**Table 1:** African horse sickness virus isolates or sequences used in this study.

AHSV strain^a^	Isolate name or number	GenBank accession number
AHSV-1 ref	HS 29/62	KF859992, AM883170
AHSV-1 field	HS 21/07	KP009629
AHSV-1 1180^b^	1180	KP009717
AHSV-2 ref	HS 82/61	KF860003
AHSV-2 field	HS 90/07	KP009637
AHSV-2 Nigeria	HS 02/07	FJ196590
AHSV-3 ref	HS 13/63	KM886360
AHSV-3 field	HS 73/08	KP009647
AHSV-3 cloned	N/A	U19881
AHSV-3 Ladysmith^b^	Ladysmith	KP009767
AHSV-4 ref	HS 32/62	KM609473
AHSV-4 field	HS 128/06	KP009659
AHSV-4 Vryheid^b^	Vryheid	KP009777
AHSV-5 ref	HS 30/62	KM886352
AHSV-5 field	HS 28/08	KP009667
AHSV-5 Westerman^b^	Westerman	KP009789
AHSV-6 ref	HS 39/63	KF860014
AHSV-6 field	HS 04/08	KP009679
AHSV-6 cloned	N/A	NC006019
AHSV-7 ref	HS 31/62	KF860024
AHSV-7 field	HS 23/08	KP009689
AHSV-7 Karen^b^	Karen	KP009757
AHSV-8 ref	HS 10/62	KF860034
AHSV-8 field	HS 29/00	KP009699
AHSV-9 ref	HS 90/61	KF860044
AHSV-9 field	HS 27/08	KP009707

^a^ In each case the first part of the strain name indicates the AHSV serotype, “ref” indicates the OIE Reference strain, “field” represents recent field isolates from South Africa, and “cloned” refers to two previously published sequences for which the isolate number is not known.

^b^ Historic neurotropic strains of AHSV.

The open reading frame (ORF) Finder tool (http://www.ncbi.nlm.nih.gov/projects/gorf/) was used to identify ORFs, and the predicted amino acid sequences of their products, within each nucleotide sequence. The Compute pI/Mw tool in the Swiss Institute of Bioinformatics ExPASy resource portal (http://web.expasy.org/compute_pi/) was used to determine the molecular mass of the predicted proteins. MAFFT (multiple alignment using fast Fourier transform) version 7.212 (http://mafft.cbrc.jp/alignment/server/) was used for sequence alignments and to construct Neighbour Joining trees with 1000 bootstrap resamplings. Sequence identity was determined using the shortest aligned length and gaps were treated as single deletion events.

### Antisera

A group-specific anti-AHSV serum, generated through immunisation of rabbits with sucrose gradient-purified intact AHSV-9 particles, has been described previously [36]. An anti-NS4 serum (GenScript) was produced in rabbits using a mixture of two antigenic peptides QGAGLEGEEWAEWL (representing residues 2–15 of clade I NS4) and MIEEWRARNLREAD (representing residues 33–46 of clade II NS4) as immunogen. A mouse monoclonal anti-fibrillarin antibody (38F3) was obtained from Abcam.

### SDS-PAGE and Western blot analysis

BSR cells were infected with AHSV at a MOI of 0.1 and harvested by low speed centrifugation at three days post infection. The cell pellet was resuspended in phosphate buffered saline (PBS, pH 7.4) and lysed by passage through a 29G needle. Bacterial cells were lysed as described below. Proteins in lysates or supernatants were denatured in protein solvent buffer (0.67 M Tris, 6.7% SDS, 10% glycerol, 5% 2-mercaptoethanol) for 5–10 minutes at 95°C and separated using 15% SDS-PAGE.

For SDS-PAGE, the separated proteins were stained with Coomassie Brilliant Blue for 15–20 minutes and destained overnight in 5% acetic acid, 5% methanol. For Western blotting, the proteins were transferred to a nitrocellulose membrane (Hybond-C Extra, Amersham Biosciences) in transfer buffer (25 mM Tris, 192 mM glycine, 20% methanol, pH 8.3). The membrane was incubated in blocking solution (1–2% fat free milk powder in PBS) followed by incubation overnight with agitation in anti-NS4 serum at a 1:50 or 1:100 dilution. After incubation with horseradish peroxidase (HRP)-conjugated protein A (1:10 000 in 1% milk, Calbiochem), the labelled protein was detected with 4-chloro-1-naphthol (Sigma Aldrich).

### Confocal microscopy

BSR cells were seeded on 12 mm diameter round coverslips in 24-well plates and infected with AHSV at a MOI of 2 or 3. Cells were fixed with 4% paraformaldehyde (PFA) and permeabilised with 0.2% Triton X-100 (Merck Millipore) in PBS. After one hour at room temperature in blocking solution (5% milk powder in PBS) coverslips were incubated overnight in primary antiserum diluted 1:100 (anti-NS4) or 1:250 (anti-fibrillarin) in 1% blocking solution. For secondary labeling, cells were incubated with Alexa Fluor-488 conjugated anti-rabbit IgG or Alexa Fluor-633 conjugated anti-mouse IgG (Invitrogen) at a 1:250 dilution. Nuclei were stained with for ten minutes using 5 mg/mL DAPI (4’,6-diamidino-2-phenylindole, Life Technologies) diluted 1:1000 in 1% blocking solution. Coverslips were mounted on microscope slides in VectaShield mounting medium (Vector Laboratories) and the edges sealed. Samples were viewed with a Zeiss LSM510 Meta Laser Scanning Confocal Microscope.

### Immunohistochemistry

Wax-embedded heart, lung and spleen tissues were obtained from AHSV-positive and-negative horses. The AHSV-positive horses presented for routine diagnostic necropsy at the Veterinary Pathology Reference Laboratory (University of Pretoria). The one horse was a one year old cross-Arabian gelding naturally infected with AHSV-4 (S1253-04) that died acutely of AHSV. The other was a three-year-old Boerperd female infected with AHSV-7 ref (HS 31/62) as part of an unrelated vaccine trial. Routine diagnostic tissue samples were collected as part of the necropsy examination and were subsequently used during this study. The use of diagnostic samples for research or teaching purposes is in accordance with the terms of the University of Pretoria's diagnostic service. The negative control tissues were previously provided by a registered veterinary pathologist in New Zealand and had been obtained from horses at an abattoir in New Zealand (where AHS does not occur). These horses had been slaughtered according to internationally prescribed humane methods for the slaughter of horses for human consumption. Ethical approval for the use of the AHSV-4 infected and uninfected horse material was obtained from the University of Pretoria, Faculty of Veterinary Science, Animal Use and Care Committee, with Protocol and Ethical Approval number V049/04, and other work based on these samples has been published previously [36,37]. Ethical approval for the use of the AHSV-7 infected material was obtained from the Deltamune Ethics Committee, Project number 201308–27, Ethics Committee reference CR-13-119.

The tissues were sectioned at a thickness of 3–4 μm and subjected to immunoperoxidase labelling procedures. Immunohistochemistry was performed by hand following validated protocols [38,39]. For the primary antibodies, the group-specific anti-AHSV serum (1:2000) and the anti-NS4 serum (1:6400) were used. All sections were routinely deparaffinised, hydrated and then incubated with 3% hydrogen peroxide in methanol for 15 minutes at room temperature to quench endogenous peroxidase activity. Thereafter, tissue sections for labelling with the group-specific serum were pre-treated with Protease XIV (Sigma) for 30 min at 37°C, while sections for labelling with the anti-NS4 serum were pre-treated by microwave heating for 14 min at 96°C in citrate buffer (pH 6.0). This was followed by incubation of sections with normal goat serum (Sigma) diluted 1:10 with PBS (pH 7.6) containing 0.1% bovine serum albumin for 20 minutes in a humidified chamber at room temperature, to prevent non-specific immunoglobulin binding. Following treatment of sections with the group-specific serum (for 45 min), sections were further incubated for 30 min with a goat-anti-rabbit link antibody (Dako, Denmark) diluted 1:150 with 10% normal goat serum, followed by application of the Vectastain ABC Kit (Vector Laboratories) according to manufacturer’s instructions. After incubation of tissue sections with the anti-NS4 serum (for 60 min), sections were further treated with the DakoREAL EnVision HRP Rabbit/Mouse Polymer Detection System (Dako, Denmark) for 30 min. For both the anti-AHSV group-specific serum and the anti-NS4 serum, development with the NovaRED substrate (Vector Laboratories) was executed for 1–2 minutes. Tissues were subsequently stained with Mayer’s hematoxylin (30 seconds) for nuclear counterstaining in all sections. Positive tissue controls included validated tissue sections (heart, lung and spleen) with known reactivity with the tested antigens. For negative reagent controls, buffer (0.1 molar PBS (pH 7.6) containing 0.1% bovine serum albumin) was substituted for the primary antiserum in each case.

### Bacterial expression of AHSV NS4

Based on the predicted NS4 amino acid sequences of the AHSV-4 field and AHSV-3 field isolates, NS4 gene sequences codon-optimised for bacterial expression were synthesized and cloned into pUC57 (GenScript) to generate the plasmids pUC57-NS4-I and pUC57-NS4-II respectively. The codon-optimised NS4 inserts were excised from pUC57 using NdeI and XhoI, and subcloned into the bacterial expression vector pStaby1.2 (Delphi Genetics) to generate pStaby-NS4-I and pStaby-NS4-II. All plasmid purifications were done using High Pure Plasmid Isolation and Genopure Plasmid Maxi Kits (Roche).

Recombinant plasmids pStaby-NS4-I and pStaby-NS4-II were transformed into *E*. *coli* BL21(DE3) cells (Novagen), which have an IPTG inducible T7 RNA polymerase gene in the form of a phage λ-DE3 lysogen. Transformed cells were used for recombinant protein expression following IPTG induction (StabyExpress T7 Kit Manual, version 1.7 Delphi Genetics). The 6xHis-tagged NS4 proteins were purified with Protino Ni-TED 1000 Packed Columns (Macherey-Nagel). Since column purification does not guarantee the removal of all contaminating proteins, no DNaseI was added to prevent interference during the downstream nucleic acid protection assays. The cells were lysed in the presence of 1 mg/mL lysozyme by passage through 22G and 27G needles. For NS4 solubility estimates, the lysate was centrifuged at 6100 x g for 40 minutes to separate the particulate (pellet) and soluble (supernatant) fractions, which were analysed with SDS-PAGE. The supernatant was cleared with a 0.2 μm filter. Total protein concentration of the first elution fraction was determined with the Bicinchoninic Acid (BCA) Assay Kit (Sigma-Aldrich), and SDS-PAGE (Novex Bolt Mini Gel system, Life Technologies) analysis with Coomassie Brilliant Blue staining was used to estimate the proportion of total eluted protein constituted by NS4. Image Lab version 2.0.1 software (Bio-Rad) was used to evaluate the purity of each sample.

### Nucleic acid protection assays

Nucleic acid protection assays were based on the method described by Belhouchet *et al*. [32]. For the DNA protection assays, 1 μg of dsDNA (BenchTop pGEM DNA Marker, Promega) in DNaseI buffer was mixed with 500 ng of purified recombinant NS4 in a volume of 9 μL and incubated at room temperature for 20 minutes. Thereafter, 2 units of DNase I (New England Biolabs) were added to the reaction, which was incubated at 37°C for 30 minutes. The enzyme was inactivated by heating the reaction to 99°C for one minute. The reactions were analysed with agarose gel electrophoresis using agarose gels containing 2% (w/v) agarose and 0.5 μg/mL ethidium bromide. RNA protection assays were performed in the same manner with the same quantities of dsRNA (dsRNA Ladder, New England Biolabs), NS4 protein and RNase III (ShortCut RNase III, New England Biolabs), but with the addition of 20 mM MnCl_2_ to the reaction. After incubation, the enzyme was inactivated by the addition of 50 mM EDTA and the reactions were analysed with 3% agarose gel electrophoresis.

## Results

### *In silico* analysis of AHSV genome segment 9 sequences

The Seg-9 nucleotide sequences of 26 AHSV isolates, representing all nine known AHSV serotypes, were analysed with the ORF Finder tool (http://www.ncbi.nlm.nih.gov/projects/gorf/) to identify ORFs and to predict the amino acid sequences of their products. The main ORF encoding VP6, as well as a second ORF encoding the putative NS4 protein, was present in all Seg-9 sequences. All but one of the predicted NS4 reading frames were in the +1 frame relative to that of the VP6 reading frame. The NS4 ORFs were usually either 435 or 465 nucleotides (nt) in length, encoding proteins of 144 or 154 amino acids (aa), respectively (Table 2).

**Table 2:** A summary of the properties of the NS4 open reading frame in AHSV Seg-9.

	Serotype	Length (nt)	Position	Reading frame	Size in kDa (aa)
**Clade I, NS4-I**	AHSV-1 field	435	214–648	+1	16.68 (144)
	AHSV-4 field	435	214–648	+1	16.77 (144)
	AHSV-4 ref	435	214–648	+1	16.69 (144)
	AHSV-5 Westerman	435	214–648	+1	16.62 (144)
	AHSV-6 field	435	214–648	+1	16.8 (144)
	AHSV-6 ref	435	214–648	+1	16.69 (144)
	AHSV-7 field	435	214–648	+1	16.78 (144)
	AHSV-8 field	435	214–648	+1	16.8 (144)
**Clade II, NS4-II**	AHSV-1 ref	465	192–656	+3	18.15 (154)
	AHSV-1 ref AM883170	465	193–657	+1	18.14 (154)
	AHSV-1 1180	324	148–471	+1	13.07 (107)
	AHSV-2 field	465	193–657	+1	18.19 (154)
	AHSV-2 Nigeria	465	193–657	+1	18.17 (154)
	AHSV-2 ref	465	193–657	+1	18.19 (154)
	AHSV-3 field	465	193–657	+1	18.2 (154)
	AHSV-3 ref	510	148–657	+1	20.09 (169)
	AHSV-3 cloned U19881	510	148–657	+1	20.09 (169)
	AHSV-3 Ladysmith	510	148–657	+1	20.09 (169)
	AHSV-4 Vryheid	492	193–684	+1	19.29 (163)
	AHSV-5 field	465	193–657	+1	18.2 (154)
	AHSV-5 ref	465	193–657	+1	18.2 (154)
	AHSV-6 cloned NC006091	432	148–579	+1	17.26 (143)
	AHSV-7 Karen	465	193–657	+1	18.02 (154)
	AHSV-7 ref	465	193–657	+1	18.23 (154)
	AHSV-8 ref	465	193–657	+1	18.15 (154)
	AHSV-9 field	465	193–657	+1	18.2 (154)
	AHSV-9 ref	465	193–657	+1	18.26 (154)

The length, position, reading frame and size of the NS4 amino acid (aa) sequences and ORF nucleotide (nt) sequences predicted with the ORF Finder tool are summarised. The suffix “ref” indicates a reference strain and the suffix “field” indicates a field isolate. GenBank accession numbers of previously published sequences are included.

**Fig 1:**
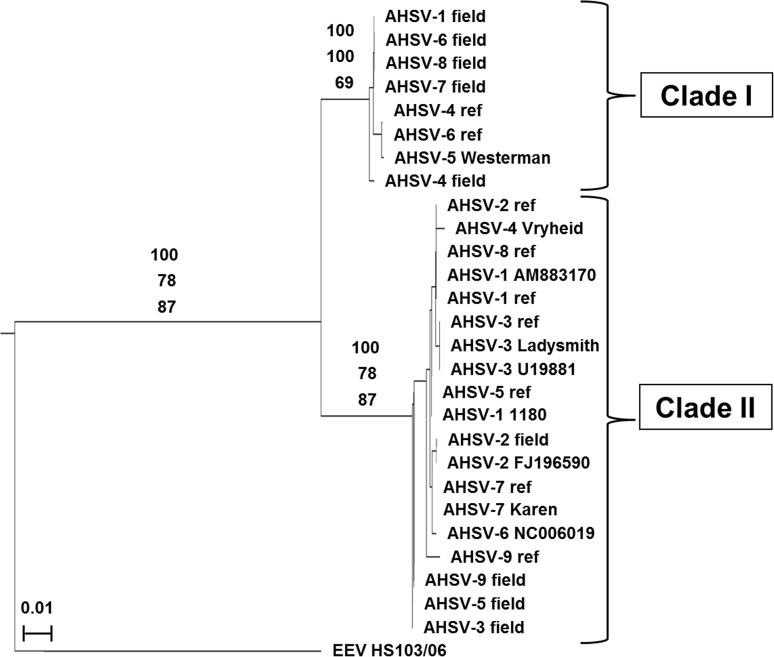
Neighbour Joining tree showing the relationships between the predicted NS4 amino acid sequences. Equine encephalosis virus (EEV HS 103/06, GenBank: FJ183392.1) was used as an outgroup. The scale bar indicates the branch length corresponding to 0.01 nucleotide changes. The suffix “ref” denotes a reference strain and the suffix “field” denotes a field isolate. The Genbank accession numbers of the previously published sequences are shown. Bootstrap values of the Seg-9 nt tree (top value), VP6 aa tree (middle value) and NS4 aa tree (bottom value) are indicated.

**Fig 2:**
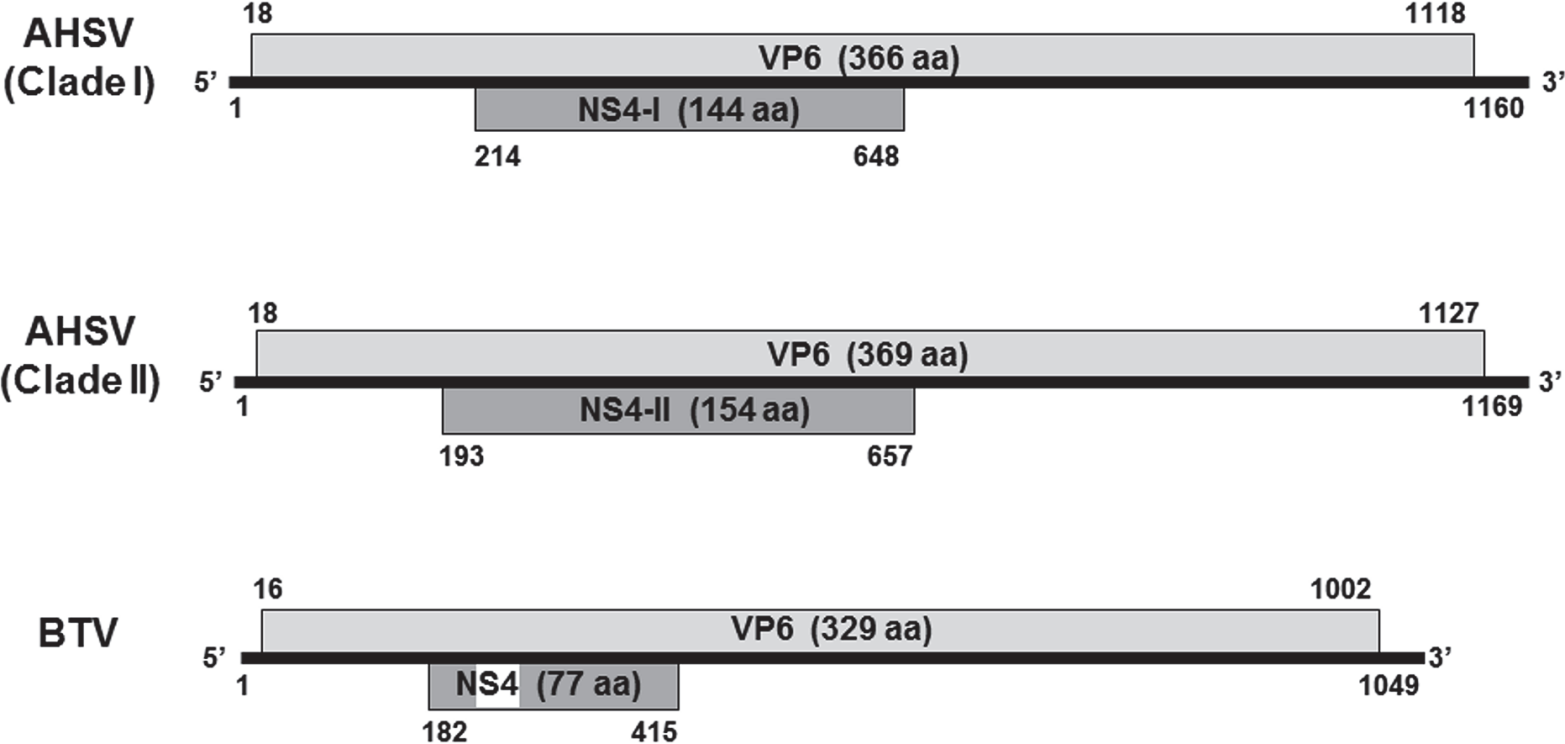
Genome maps of Seg-9 of AHSV and BTV. Seg-9 maps showing the nucleotide positions of the VP6 open reading frame (ORF) (light gray) and the NS4 ORF (dark gray) that were most common in each clade. AHSV groups into two different clades, with the Clade I VP6 (366 aa) and NS4 (144 aa) coding sequences being slightly smaller than those of Clade II VP6 (369 aa) and NS4 (154 aa). A nuclear localisation signal (NLS) (white block) is present at residues 12–24 of BTV NS4, but absent from most AHSV NS4 sequences.

Seg-9 nucleotide and predicted VP6 and NS4 amino acid sequences were compared using MAFFT (multiple alignment using fast Fourier transform) and their relationships visualised with Neighbour-Joining trees. In each case, the isolates grouped into two main clades. The same isolates were always seen in the same clade, regardless of whether nucleotide or amino acid sequences were analysed. These two groups were designated Clade I and Clade II (Table 2 and Fig 1). The lengths and sequences of Seg-9, VP6 and NS4 (and their ORFs) differed between these clades. Higher levels of sequence identity were seen within clades, with lower identity between clades (Table 3). The Seg-9 nucleotide (65% identity) and VP6 amino acid (52% identity) sequences were less conserved among 24 AHSV isolates (Table 3) than predicted previously (97% Seg-9 identity and 95% VP6 identity), where only AHSV-3 (U19881) and AHSV-6 (NC006019.1) were available for comparison [28]. NS4 amino acid sequences showed only 52% identity between Clade I and Clade II. Furthermore, the predicted nuclear localisation signals (NLS) previously described by Belhouchet *et al*. [32] were absent from most of the putative NS4 proteins identified during this study. Only AHSV-1 1180, AHSV-3 ref, AHSV-3 Ladysmith, AHSV-3 cloned and AHSV-6 cloned contained the NLS sequences; their predicted NS4 start codons are 45 nucleotides upstream of all other Clade II NS4 start sites and the predicted NLS is found within the extra 15 N-terminal residues present only in these strains. Based on this information, the types of NS4 produced by members of each clade were designated NS4-I (Clade I) and NS4-II (Clade II), respectively. A schematic representation comparing the Seg-9 gene maps of AHSV to that of BTV [30] is shown in Fig 2.

**Table 3:** A summary of the percentage sequence identity for AHSV Seg-9, VP6 and NS4 within and between Clades I and II.

	Sequence				
Group	Segment 9 nt	VP6 ORF nt	VP6 aa	NS4 ORF nt	NS4 aa
**Between clades** ^**a**^	64.7	63.4	51.8	57.9	52.0
**Within Clade I**	94.1	93.9	91.8	94.0	93.1
**Within Clade II** ^**a**^	86.4	86.0	78.3	83.9	80.8

The full genome segment 9 nucleotide (nt) sequences, as well as VP6 and NS4 ORFs and amino acid (aa) sequences, were compared.

^a^ AHSV-6 (NC006019) and AHSV-1 (1180) were omitted from the analyses because their predicted NS4 proteins are considerably shorter than those of all other isolates.

### Expression and intracellular localisation of AHSV NS4

**Fig 3:**
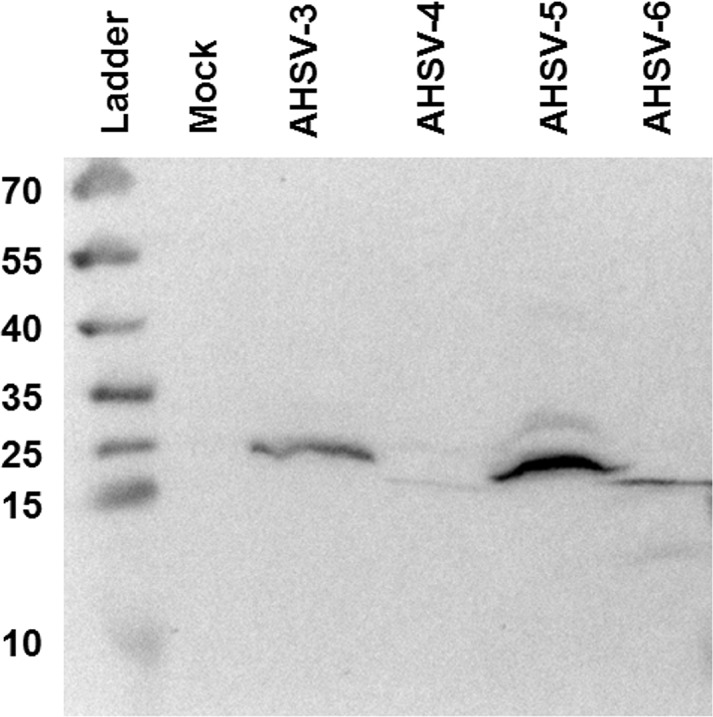
Western blot with anti-NS4 demonstrating the presence of NS4 in AHSV-infected BSR cells. Cell lysates of mock infected cells or cells infected with AHSV-3 AHSV-4, AHSV-5 or AHSV-6 were subjected to Western blotting using the anti-NS4 serum. Sizes of the molecular weight marker are indicated in kDa.

In order to determine whether the predicted NS4 protein is expressed in AHSV infected cells, lysates from BSR cells infected with AHSV-3 ref (Clade II), AHSV-4 ref (Clade I), AHSV-5 ref (Clade II) or AHSV-6 ref (Clade I) were analysed with Western blotting using the anti-NS4 serum. Unique bands were detected in all infected samples (Fig 3). These bands were approximately 20 kDa in size for Clade I viruses, and 23 kDa for Clade II viruses, in both cases slightly more than the predicted molecular weights. These results confirmed that both types of putative AHSV NS4 protein are expressed in mammalian cells.

**Fig 4:**
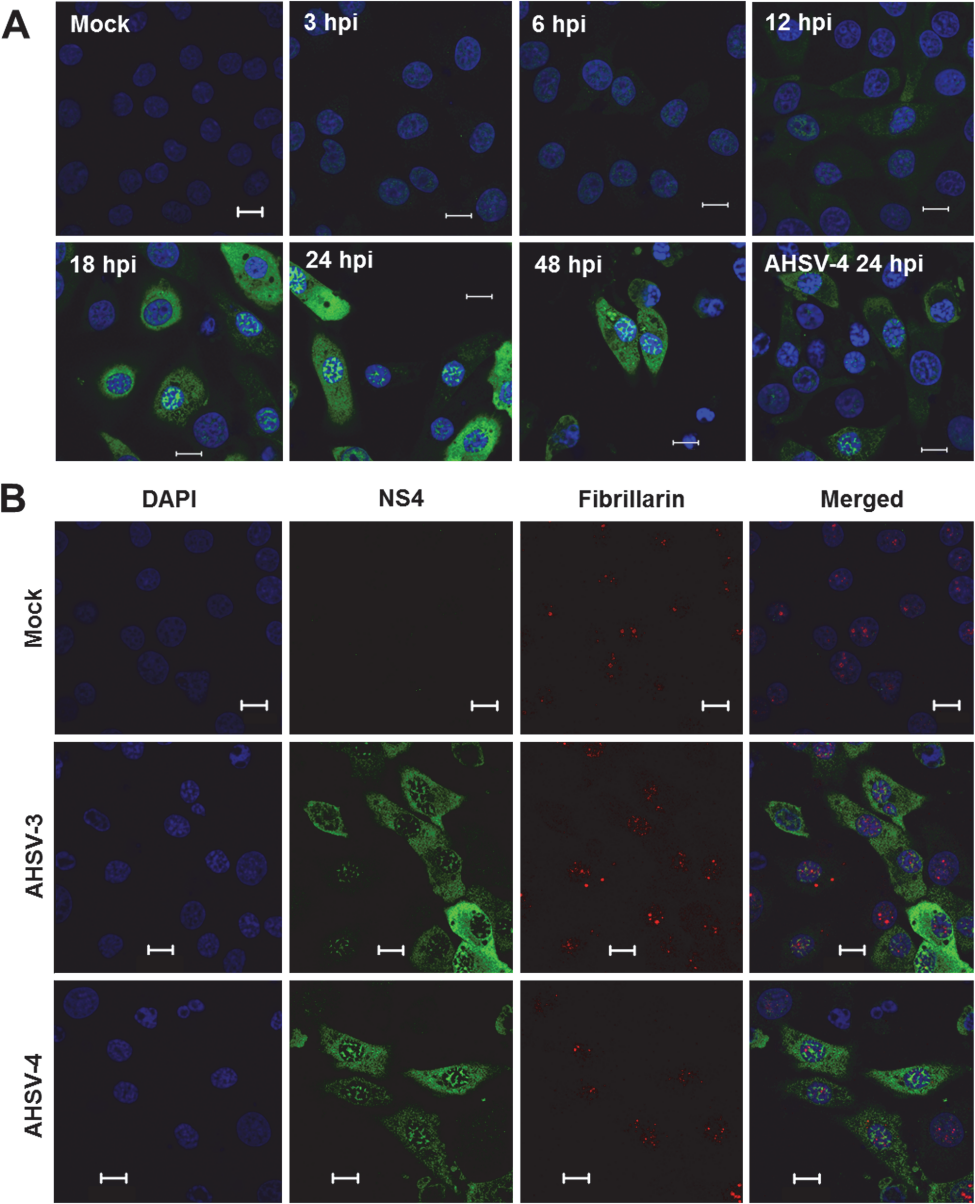
Confocal micrographs showing NS4 expression in BSR cells infected with different serotypes of AHSV. **A:** Cells were infected with AHSV-3, immunolabelled with anti-NS4 and Alexa-Fluor 488 IgG (green) and viewed at 3–48 hpi. NS4 had a similar distribution in cells infected with AHSV-4 (bottom right). **B:** Cells were mock infected (top panel) or infected with AHSV-3 (middle panel) or AHSV-4 (bottom panel) and labelled with anti-NS4 and Alexa Fluor 488 IgG, and anti-fibrillarin and Alexa Fluor 594 (red) at 24 hpi. Nuclei were stained with DAPI (blue).

In order to study the intracellular localisation of NS4 within AHSV infected cells, BSR cells were infected with AHSV-3 (representing an NS4 protein from Clade II, or NS4-II) or AHSV-4 (representing NS4-I) and labelled with anti-NS4 for confocal microscopy at 3, 6, 12, 18, 24 and 48 hours post infection (hpi) (Fig 4A). Small amounts of NS4 could be detected by 3 hpi, with a diffuse distribution in the nucleus and cytoplasm. By 12 hpi, small punctate to stellate foci were also observed in the nucleus, while the cytoplasmic distribution remained predominantly diffuse. The same distribution was observed at 24 hpi and persisted until 48 hpi, with an increase in the intensity of NS4 labelling over time (Fig 4A). No NS4 was observed at the plasma membrane in cells infected with AHSV-3 or AHSV-4. No overt differences were observed in the distribution of the two types of NS4.

Since the punctate distribution of AHSV NS4 in the nucleus resembled that of BTV NS4, which has a nucleolar localisation, AHSV infected BSR cells were subjected to co-immunolabelling of NS4 and the nucleolar marker fibrillarin at 12, 24 and 48 hpi in order to determine whether AHSV NS4 also localises to the nucleolus. Cells labelled with anti-fibrillarin showed distinct fluorescent foci in the nucleus (Fig 4B) that were similar to those described by Belhouchet *et al*. [32] and Ratinier *et al*. [33]. Labelling of fibrillarin resulted in weaker fluorescence at later time points after infection. This was also reported for BTV and was proposed to result from cell death-related nuclear events induced by viral infection [32]. Although both fibrillarin and NS4 could be detected simultaneously in the nucleus, they did not co-localise. This suggests that AHSV NS4 does not occur in the nucleolus. Similar labelling of NS4 and fibrillarin was done using cells infected with AHSV-2 (Clade II, at 48 hpi), AHSV-5 (Clade II, at 12 and 24 hpi), AHSV-6 (Clade I, at 48 hpi), AHSV-8 (Clade II, at 24 hpi), which in essence all gave similar results to those depicted here (not shown).

**Fig 5:**
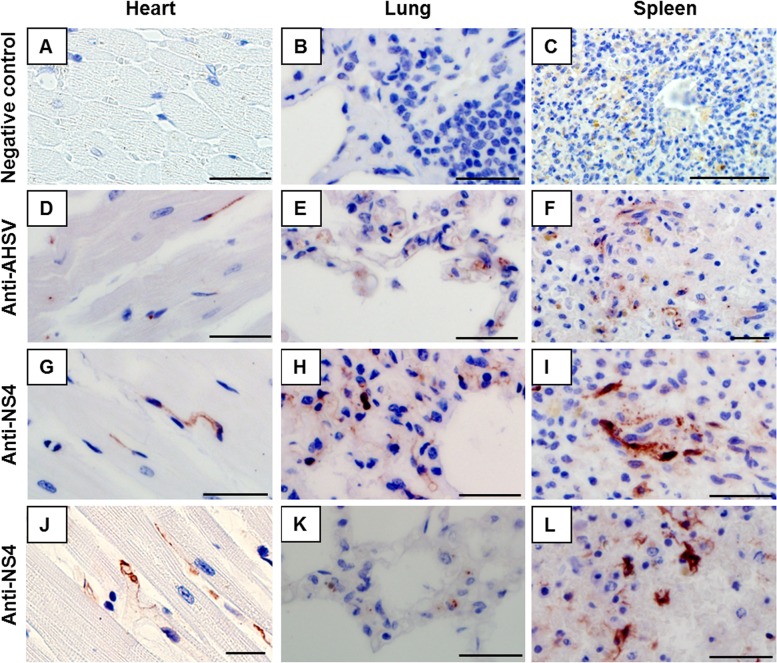
Immunohistochemistry of AHSV-infected horse tissues. Immunoperoxidase labelling indicating the localisation of NS4 (red) predominantly within the cytoplasm and/or nucleus of microvascular endothelial cells and mononuclear phagocytes in heart, lung and spleen tissue of horses infected with AHSV-4 (D-I) or AHSV-7 (J-L). NS4 was also observed in stellate-shaped cells with long cytoplasmic processes in the red pulp of the spleen (L). Tissues from uninfected horses labelled with anti-NS4 antibody were used as negative controls (A-C), and AHSV-4 infected tissues stained with a polyclonal anti-AHSV serum as positive controls (D-F). Scale bars correspond to 20 μm.

In naturally infected horses, it was previously shown that sections of heart, lung and spleen consistently label positive for AHSV proteins across all serotypes [36,37]. Here, tissues from confirmed (virus isolation- and virus neutralisation or RT-PCR-positive) AHSV-4 (probably Clade I, but not confirmed with sequencing) and AHSV-7 (Clade II) cases (naturally and experimentally infected horses, respectively, that presented for necropsy) were selected for immunoperoxidase labelling to determine the location of NS4 within the mammalian host of AHSV (Fig 5). The group-specific anti-AHSV serum used as positive control predominantly labelled microvascular endothelial cells and monocyte-macrophages in sections of heart, lung and spleen. The pattern of positive control labelling varied from fine dust-like to coarse granules, which occurred singly or in small clusters in the cytoplasm of target cells (Fig 5D–5F). Labelling with the anti-NS4 serum, on the other hand, was not only cytoplasmic and granular, but also diffusely and strongly nuclear (Fig 5G–5L). In both AHSV-4 (Fig 5G–5I) and AHSV-7 (Fig 5J–5L) infected horses, the target cells where NS4 expression was detected included microvascular endothelial cells, especially in heart and lung, and stellate-shaped dendritic macrophage-like cells with long cytoplasmic processes particularly located in the red pulp of the spleen (Fig 5L). There was no positive labelling in the tissue sections (heart, lung and spleen) from the negative-tissue control horses from New Zealand (Fig 5A–5C) or in the negative reagent controls (not shown).

### Nucleic acid binding activity of AHSV

**Fig 6:**
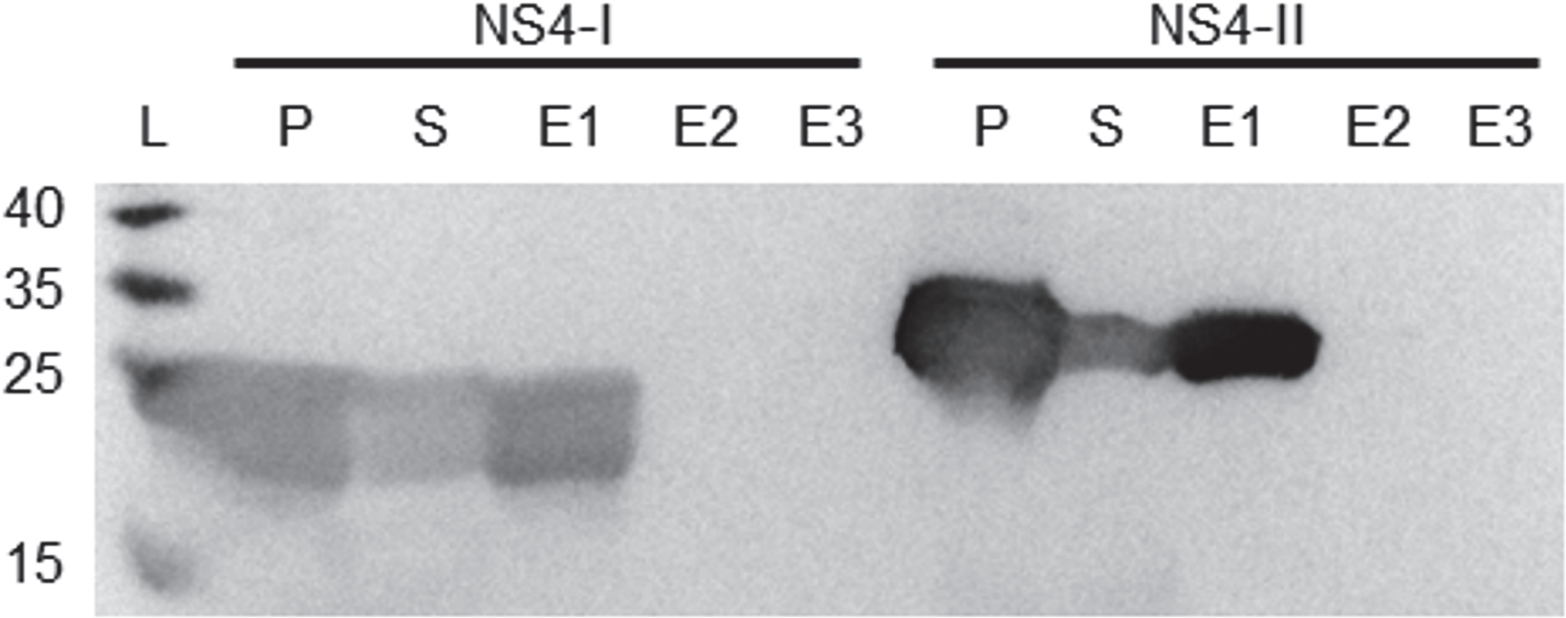
Western blot of pellets, supernatants and elution fractions of bacterial cells expressing NS4. The pellet (P) and supernatant (S) of *E*. *coli* BL21(DE3) cells expressing NS4-I or NS4-II were analysed with Western blotting using the anti-NS4 serum. Soluble NS4 in the supernatant was subjected to affinity purification and the three elution fractions analysed (E1-E3). L: Size ladder (kDa).

Previous bioinformatic analyses of NS4 sequences [32] suggested that AHSV NS4 has nucleic acid binding or modifying activity. To investigate this property, codon optimised His-tagged NS4-I and NS4-II proteins were expressed in *E*. *coli*. The cell lysates from these cells were centrifuged and fractions analysed with SDS-PAGE (not shown) and Western blotting, which showed that although the majority of NS4 was found in the pellet using this extraction method, some NS4 occurred in soluble form in the supernatant (Fig 6). Following column purification of the soluble fraction, most NS4 was present in the first elution fraction (Fig 6), and the eluate was relatively pure, containing 300–400 μg/ml NS4 as determined with SDS-PAGE and the BCA assay.

**Fig 7:**
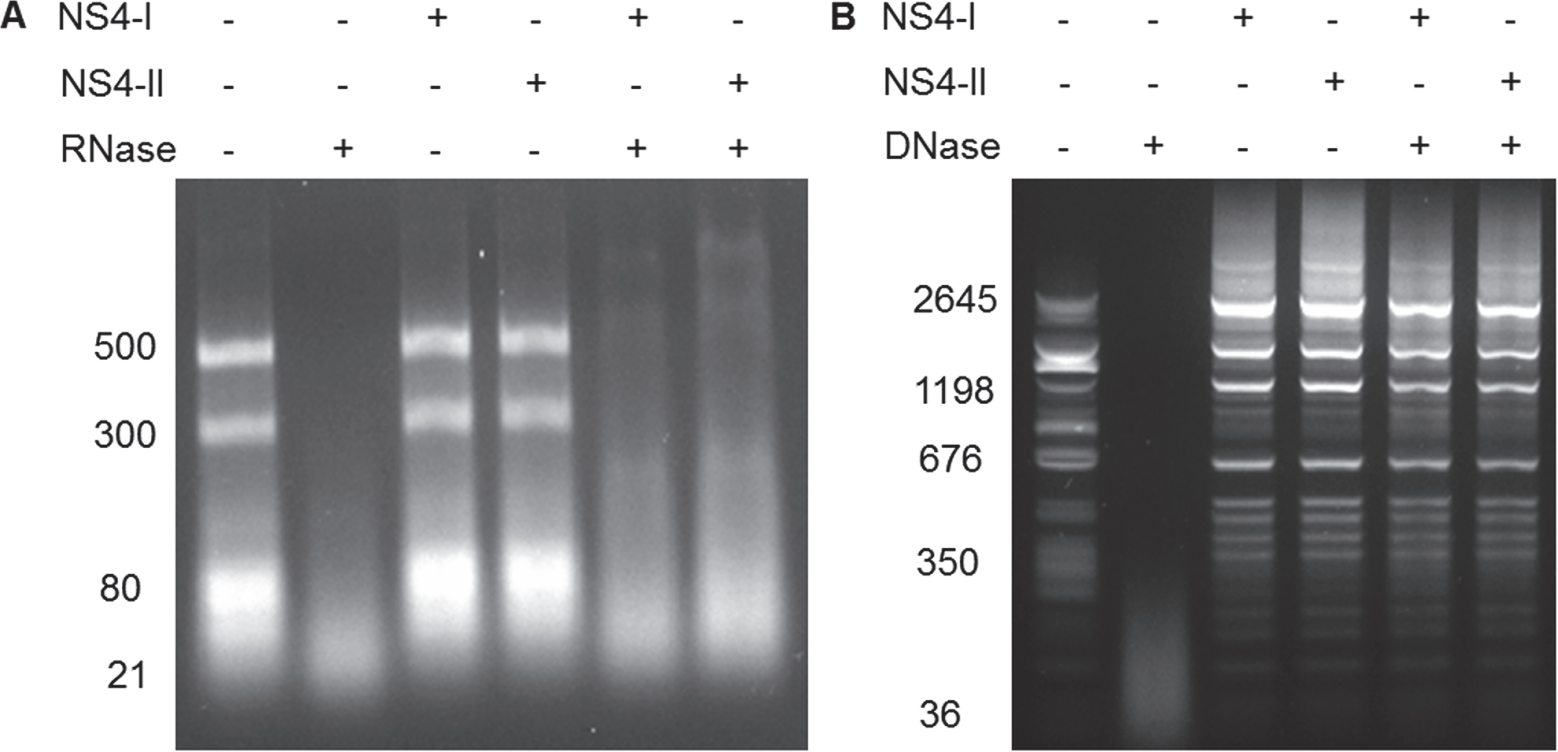
Nucleic acid protection assays. Agarose gel electrophoretograms depicting NS4 RNAse (A) and DNAse (B) protection assays. The nucleic acid ladders were incubated with (+) or without (-) purified NS4-I or NS4-II protein and nucleases as indicated in the top panel.

To determine whether AHSV NS4 binds dsRNA, a dsRNA ladder was incubated with 500 ng of purified recombinant NS4-I or NS4-II prior to treatment with RNaseIII. RNase III digestion of dsRNA yields fragments of 18–21 bp. Incubation of dsRNA with RNase III resulted in degradation, while incubation of dsRNA with NS4 alone did not affect the integrity of the RNA (Fig 7A). Pre-incubation of dsRNA with NS4 prior to treatment with RNase III did not protect the dsRNA from degradation (Fig 7A). This suggests that NS4 does not bind dsRNA non-specifically *in vitro*. A nuclease protection assay using a dsDNA ladder and DNaseI was also performed. DNase I cuts DNA or DNA:RNA hybrids non-specifically to produce di-, tri-, and oligonucleotide fragments. During the dsDNA protection assay, incubation of dsDNA with DNase I resulted in degradation and incubation with either type of NS4 alone did not affect DNA integrity. No degradation was observed when dsDNA was incubated with NS4 prior to DNase treatment (Fig 7B). This suggests that both NS4-I and NS4-II can bind dsDNA *in vitro*. These results confirm the predicted nucleic acid binding properties of AHSV NS4, and correspond to what was observed for BTV NS4, which also binds dsDNA but not dsRNA.

## Discussion

Recently, Firth [30] showed that most orbiviruses are expected to have an additional ORF within the genome segment encoding the minor structural protein VP6. Belhouchet *et al*. [32] and Ratinier *et al*. [33] subsequently demonstrated that this putative protein is expressed in cells infected with BTV or GIV and named it NS4. BTV and GIV NS4 exhibited nuclear, nucleolar, cytoplasmic and plasma membrane localisation and associated with cytoplasmic lipid droplets [32]. Furthermore, although not essential for viral replication *in vivo* or *in vitro*, BTV-8 NS4 appears to modulate the host innate immune response in interferon-induced cells [33].

The aim of this investigation was to characterise the properties of AHSV NS4. Nucleotide sequences of 26 AHSV isolates, representing all nine known AHSV serotypes, were examined. The predicted NS4 ORF was present in all of these isolates. A larger amount of variation than previously reported was observed between the AHSV Seg-9 nucleotide sequences, as well as in the VP6 and NS4 ORFs and predicted proteins. Two main types of NS4 that differ in length and amino acid sequence appeared to exist amongst AHSV serotypes. These proteins were designated NS4-I and NS4-II.

Immunoblotting revealed that the putative NS4-I and NS4-II proteins were expressed in AHSV-infected mammalian cells. The NS4 proteins detected with immunoblotting were slightly larger than predicted. The reason for this discrepancy is unclear, but it may not be significant. A slight difference in the predicted and visualised size with Western blotting was also reported for BTV NS4 [32]. The intracellular localisation of NS4 was examined with confocal microscopy. The proteins localised to both the nucleus and cytoplasm of AHSV infected cells. Interestingly, the NS4 amino acid sequences of all the AHSV isolates (except AHSV-3 ref) used for immunocytochemistry lacked the N-terminal region predicted to contain nuclear localisation signals [30], suggesting that AHSV NS4 is trafficked to the nucleus by a different, or multiple, mechanisms. The presence of a classical NLS is not a requirement for trafficking to the nucleus. For example, non-classical NLS have been identified [40,41] and proteins smaller than 40 kDa may enter the nucleus via passive diffusion [42]. NS4-I and NS4-II were detected in both the nucleus and cytoplasm from 3 hpi. NS4 was diffusely distributed or formed punctate foci within the nucleus of infected cells and was distributed more or less homogeneously within the cytoplasm. AHSV-4 NS4-II was detected less effectively during immunoblotting and immunolabelling. Several explanations could account for this observation. For each type of NS4, an antigenic peptide with predicted immunogenicity and high conservation within the clade was selected, and the two peptides were pooled for antibody production. These peptides may not be equally immunogenic or the antibodies may not be equally sensitive. The NS4-I antibodies detect a region that is relatively conserved amongst all serotypes, while the NS4-II antibodies detect a less conserved region. Therefore, it is possible that NS4-I antibodies detect both types of NS4 with greater sensitivity than the NS4-II antibodies. Finally, NS4-I may be expressed at lower levels than NS4-II; further experiments will be required to determine whether this is the case. Cells infected with AHSV serotypes belonging to different NS4 clades (NS4-I and NS4-II only have 52% aa identity) had a similar intracellular NS4 distribution, suggesting that the amino acid sequence differences between the two types of NS4 do not greatly affect their intracellular distribution. The punctate distribution of AHSV NS4 within the nucleus resembled that of BTV NS4 [32,33], which localised to the nucleolus. However, although the nucleolar protein fibrillarin and AHSV NS4 both formed punctate foci within the nuclei of infected cells, they did not co-localise, suggesting that AHSV NS4 does not localise to the nucleolus in these cells.

This is the first study where the localisation of NS4 within an orbivirus-infected mammalian host was investigated. NS4 was detected in heart, lung and spleen tissue of naturally and experimentally infected horses. Similar to what was observed in AHSV-infected cell monolayers, NS4 in horse tissue also occurred in the cytoplasm and nuclei of microvascular endothelial cells (primarily in the heart and lung) and stellate-shaped cells with long cytoplasmic processes (mainly in the red pulp of the spleen). These stellate-shaped cells may be dendritic macrophage-like antigen-presenting cells. These results suggest that NS4 could be of functional importance during the host-virus interaction. In particular, its apparent presence in cells with putative antigen-presenting cells within the spleen may indicate an interaction with the host immune system during infection and would be worthy of further investigation. BTV NS4 appeared to modulate host innate immunity [33]. It would be interesting to see whether this is a conserved function of orbivirus NS4 and whether it has additional roles during replication. It would also be interesting to determine whether functional differences exist between the two types of AHSV NS4.

BTV NS4 binds dsDNA non-specifically *in vitro*, whilst GIV NS4 binds both dsDNA and dsRNA. We showed that both types of AHSV NS4 bind dsDNA non-specifically, but not dsRNA. The fact that both types of AHSV NS4 can bind DNA suggests that their amino acid sequence differences do not affect nucleic acid binding activity. While the functional significance of the observed nucleic acid binding activity is not yet clear, it could clearly be important during the viral replication cycle if future work demonstrates an interaction between NS4 and host nuclear DNA. Although it is not fully understood why certain cytoplasmic viruses produce proteins that localise to the nucleus, it has been proposed that this trafficking could result from sequestration of a viral protein from the cytoplasm to reduce virus replication. An alternative explanation could be that the viral protein is targeted to the host nucleus to interfere with cellular processes to promote viral replication [43].

We have characterised the NS4 gene and amino acid sequences of 26 AHSV isolates, demonstrated that two types of AHSV NS4 are expressed, occur in the nucleus and cytoplasm of infected cultured and host mammalian cells, and bind dsDNA *in vitro*. Generally, our findings were consistent with the properties predicted by Firth [30] and Belhouchet *et al*. [32], and further confirm that the NS4 ORF occurs in all midge-borne orbiviruses studied to date. AHSV NS4 shows many similarities to BTV and GIV NS4. However, AHSV NS4 is less conserved among serotypes than BTV NS4, is intermediate in size between BTV (10 kDa) and GIV (22.5 kDa), does not localise to the nucleolus, plasma membrane or cytoplasmic lipid droplets like BTV and GIV, mostly lacks the NLSs present in BTV and GIV, and does not share the dsRNA binding activity of GIV NS4 [32,33]. Future studies will be required to determine the subnuclear localisation of NS4, identify possible interactions between NS4 and host proteins, determine its role within the AHSV insect vector, and ultimately to establish its function in the orbivirus replication cycle. If this fourth non-structural protein is indeed present in most orbiviruses, future studies of its function could cast light on an entirely new aspect of orbivirus biology. This study provides a basis for the functional characterisation of AHSV NS4, perhaps by means of a reverse genetics approach.
